# A Resident-Led Innovation in Communication: Introducing WhatsApp Communities in a Large Internal Medicine Residency Program

**DOI:** 10.7759/cureus.86754

**Published:** 2025-06-25

**Authors:** Aabid Mohiuddin, Fawaz Hussain, Astha Saini-Goeman, Mohamed Alrayyashi, Shaheena Raheem, Diane L Levine

**Affiliations:** 1 Department of Internal Medicine, Detroit Medical Center/Wayne State University, Detroit, USA

**Keywords:** communication, graduate medical education, quality improvement, whatsapp, whatsapp communities

## Abstract

Introduction

Effective communication is critical in healthcare. Despite the availability of secure messaging applications, widespread adoption has lagged due to costs and clinician preferences. WhatsApp is a popular free messaging platform that is frequently used in healthcare settings, although its use raises concerns regarding the safety of patient health information. In recent years, WhatsApp has launched a feature called WhatsApp Communities (WAC), which allows for structured communication in large groups. We identified this as a valuable tool to improve communication and collaboration in our internal medicine residency program.

Methods

At the start of the new academic year, we transitioned from a single WhatsApp group chat to a WAC platform with multiple sub-group chats. After six months, we surveyed residents using a five-point Likert scale to assess satisfaction with the new platform. We also defined and measured instances of "undifferentiated patient-care notifications" (messages sent to all residents but only truly intended for a specific cohort of residents) and compared how often they occurred in the legacy single chat versus the new WAC platform. Finally, we implemented an intervention to eliminate inappropriate sharing of protected patient health information on the platform.

Results

Approximately half (57 (47.5%)) of all residents completed the survey. Satisfaction was high: 48 (84%) were satisfied with the new platform’s organization, 49 (87.5%) with its timeliness, and 54 (95%) with its ability to keep them informed. Thirty-four (92%) residents who had used the prior single group chat preferred the newer platform. "Undifferentiated patient-care notifications" were reduced by 97% (from 204 instances to six). A chief medical resident (CMR)-led intervention yielded a statistically significant decline in the inappropriate sharing of patients’ protected health information (PHI).

Conclusion

WAC significantly improves communication in a large internal medicine residency program. It is an effective, zero-cost communication platform, which makes it especially attractive to programs with limited resources. The sharing of PHI remains a concern but can be effectively addressed through targeted orientation and moderation by CMRs.

## Introduction

The importance of effective communication in healthcare is well-known both in relation to patient care and medical education [1,2]. With regard to patient care, studies have shown that improvements in physician-physician communication are associated with improved patient outcomes; the opposite has also proven true, with one study reporting that up to 80% of medical errors are due to failures in communication [3,4].

In addition to optimizing patient care, effective communication is crucial to the delivery of medical education, especially in the clinical setting where teaching occurs outside of scheduled classroom lectures. In one study, 88% of surgery residents and 71% of attending surgeons used text messaging to discuss matters related to patient cases including both logistical details as well as feedback [5]. A separate study reported that attending physicians sharing clinical pearls via group messaging served as an effective supplement to bedside teaching and received enthusiastic feedback from residents [6].

In large academic hospitals where residents assume responsibility for patient care while also receiving ongoing medical education, facilitating communication between residents as well as between residents and faculty is important. Optimizing access to necessary information (both patient care- and medical education-related) has also been correlated with greater resident satisfaction [7].

With the importance of communication in healthcare being well-established, the strengths and limitations of different communication platforms have been increasingly studied. Given the privacy standards set by the Health Insurance Portability and Accountability Act (HIPAA), there has been a growing emphasis on balancing efficiency with security [8]. Several secure messaging applications have been developed to address this need; however, widespread adoption has been slow, with only 7% of all clinicians in a 2017 national cross-sectional survey stating that secure messaging was widely used at their hospital [9]. While these platforms are secure and often built into the native Electronic Health Record (EHR) system, hospitals must pay extra licensing costs to access them (which can be a barrier to adoption) and their accessibility across different clinical applications remains limited [10,11].

The adoption of secure messaging has also been hampered by clinicians’ individual preferences. In a qualitative study of barriers to adoption, respondents cited the burden of downloading additional applications to their devices, lack of trust in functionality, and low adoption among their peers. Additionally, respondents frequently complained that secure messaging apps were inferior to standard messaging apps, which many were already using [12].

WhatsApp is among the popular standard text messaging platforms utilized by clinicians today, due to its easy accessibility across different smartphone operating systems. It has increasingly gained popularity among clinicians over traditional methods of communication such as paging because of its capability for real-time messaging, multimedia sharing, and group messaging. In a study investigating attitudes toward its use in clinical settings, a majority of healthcare professionals (68%) perceived its use as beneficial with particular emphasis on the speed and efficiency of information exchange [13]. Despite these benefits, concerns over data privacy have prompted many to caution healthcare professionals about the sharing of patients’ protected health information (PHI) on the app. As a subsidiary of Meta, the parent group that also owns Facebook, WhatsApp does not abide by HIPAA [14].

In 2022, WhatsApp launched a new feature within its application called WhatsApp Communities (WAC). This feature enhanced the app’s functionality by allowing multiple group chats to be linked under a single umbrella, facilitating coordinated communication across large organizations with multiple sub-teams. In the context of large residency programs, WAC allows for organized collaboration between residents, fellows, and various clinical teams [15].

We implemented the use of WAC in our Internal Medicine Residency Program. By highlighting both the benefits and potential drawbacks, we aimed to demonstrate how a readily available free technology can improve communication and resident satisfaction within an internal medicine residency. We also intended to offer insights to similar programs, especially those with limited financial resources seeking to improve internal communication and collaboration without incurring significant costs.

## Materials and methods

In previous years, our program conducted real-time resident-to-resident collaborative communication via a single WhatsApp group chat, which included all internal medicine residents. Residents in our program rotate through several hospitals and clinical sites every two to four weeks. With over 120 residents, keeping track of who was working at each location was a consistent challenge.

Due to the perceived inefficiency of the numeric paging system, residents generally preferred WhatsApp to communicate with their colleagues as it allowed for two-way communication. Thus, the legacy single group chat functioned like a virtual public square where residents would share information in hopes of reaching the intended recipient. For example, a resident at the outpatient clinic may message in the group chat: “Whoever was on-call at ‘hospital A’ yesterday, I am following up on a discharged patient and wanted to ask you a question, please message me.” In other cases, the group was used for broadcast messaging, such as “There is a lecture on management of heart failure exacerbation today in ‘conference room A’, please try your best to attend!”

Unfortunately, this system faced its own limitations. Due to its undifferentiated nature, residents were alerted to each new message despite the majority being irrelevant to them. In response to unwanted distractions, many residents would then mute the notifications, thereby resulting in them missing subsequent communications that were relevant.

**Figure 1:**
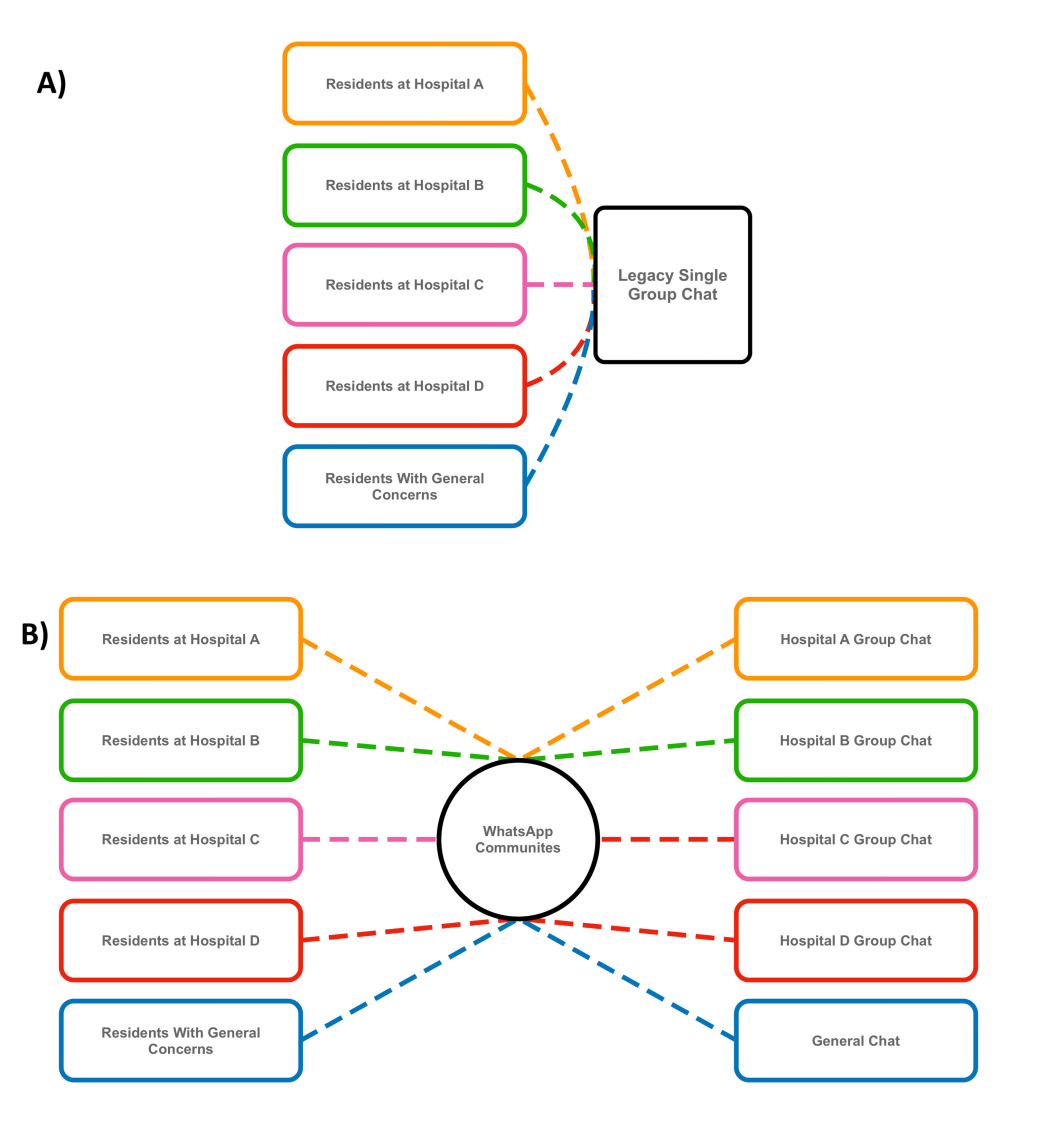
A diagram explaining the differences in communication patterns between the previous single group chat and the new WhatsApp Community. A) In the prior system, all messages between residents were sent to a single undifferentiated group chat. B) In the new WhatsApp Communities platform, messages are directed to individual group chats within the community. This ensures messaging reaches the intended recipient and allows uninvolved residents to mute notifications for rotations that they are not on. Image created by Aabid Mohiuddin, DO.

To address this shortcoming, we created a new WAC for the internal medicine program, with multiple group chats under its umbrella (Figure 1). These included dedicated chats for each of our four ward services, three intensive care unit services, the night float service, and the procedures service. An additional general-purpose group chat was created for any discussions unrelated to patient care or otherwise uncategorized. The Institutional Review Board (IRB) was consulted and determined that the project was considered quality improvement and, therefore, did not require IRB approval. At the start of the academic year, the legacy single group chat was retired, and a new communication platform was launched.

**Figure 2:**
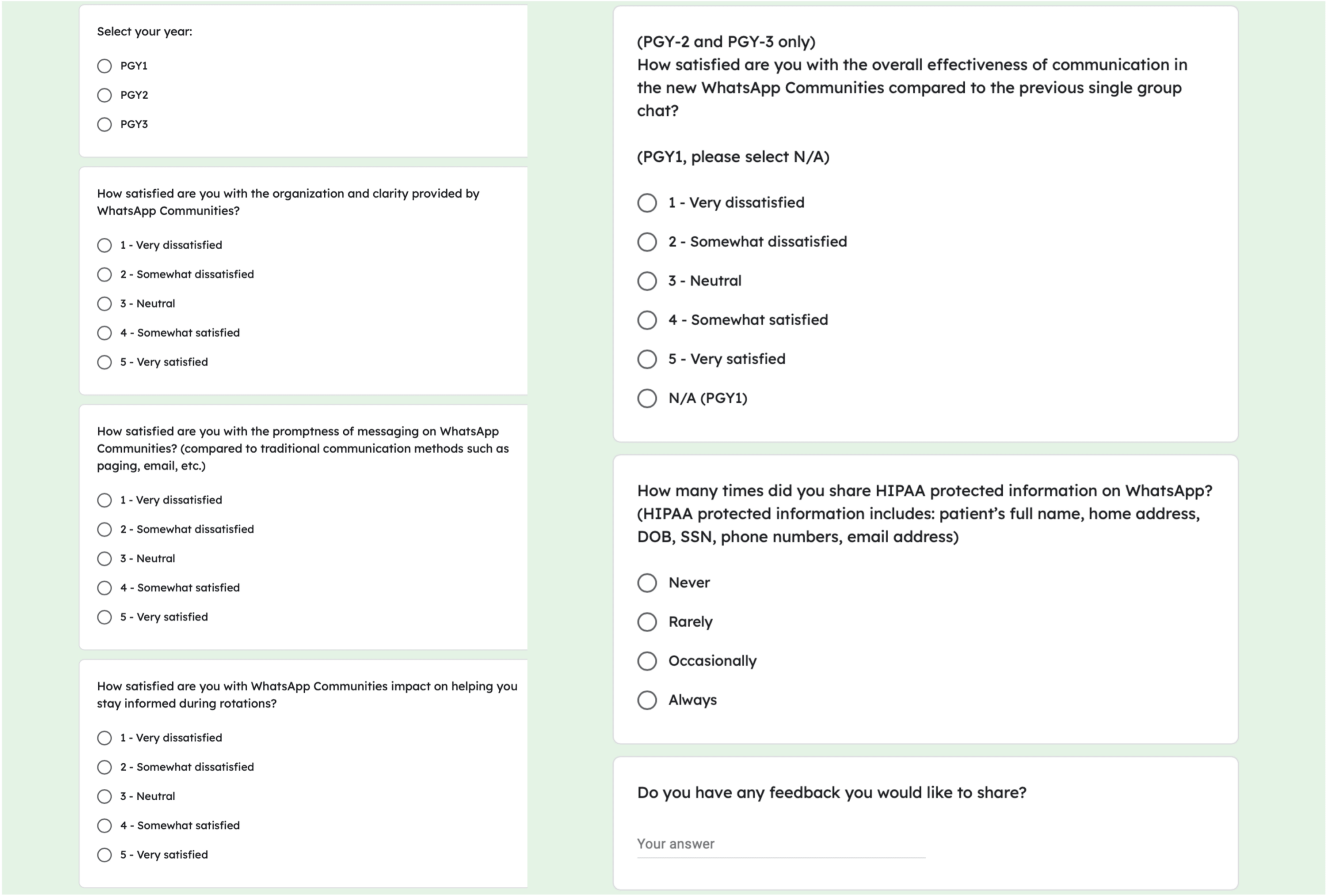
Six-month post-implementation survey of WhatsApp Communities, distributed to all residents in the internal medicine program.

Six months after implementing the WAC, we surveyed all residents in the internal medicine program. The survey was developed by the WAC-project leadership and utilized a five-point Likert Scale to evaluate resident satisfaction with communication using WAC focusing on three aspects: organization, timeliness of critical messaging, and the ability to stay informed. Surveys were sent to all residents via email as well as the WAC itself with instructions to submit within a five-day timeframe. It consisted of five questions, with a sixth question allowing for comments. Residents were required to denote whether they were in their first, second, or third year of postgraduate training. Those who had previously used the legacy single group chat (second- and third-year residents) were asked to compare their satisfaction with the new platform to the prior system. A screenshot depicting the survey questions is shown below in Figure 2.

We also performed an objective review and comparison between the legacy single group chat and the WAC system to evaluate how effectively the new platform reduced irrelevant messaging. This was done by analyzing the frequency of "undifferentiated patient-care notifications" over comparable 90-day periods. "Undifferentiated patient-care notifications" were defined as messages intended for a specific resident or cohort but instead broadcast to all 120 categorical internal medicine residents. Typical examples included messages like “Who is on call at ‘Hospital A’?,” “Who is managing the patient in ‘Room X’?,” or “To the resident managing ‘Room X’, please call ‘Y’.” We also included cases where an alphanumeric page meant for a covering resident was mistakenly sent to a non-covering resident and then reposted on the platform to reach the appropriate individual.

Finally, we asked residents to self-report how often they shared PHI on the WAC, via a survey question. PHI was defined as any of the following: patient’s full name, home address, date of birth, social security number, phone number, or email address. When results revealed that many residents admitted to sharing PHI, we implemented an intervention aimed at eliminating these incidences. Chief medical residents (CMRs) began sending periodic, program-wide reminders about the appropriate sharing of clinical information and also contacted individual residents directly to alert them of the violating messages. To evaluate the impact of these efforts, we tracked instances of PHI sharing on the WAC during the seven days before and after intervention.

## Results

**Figure 3:**
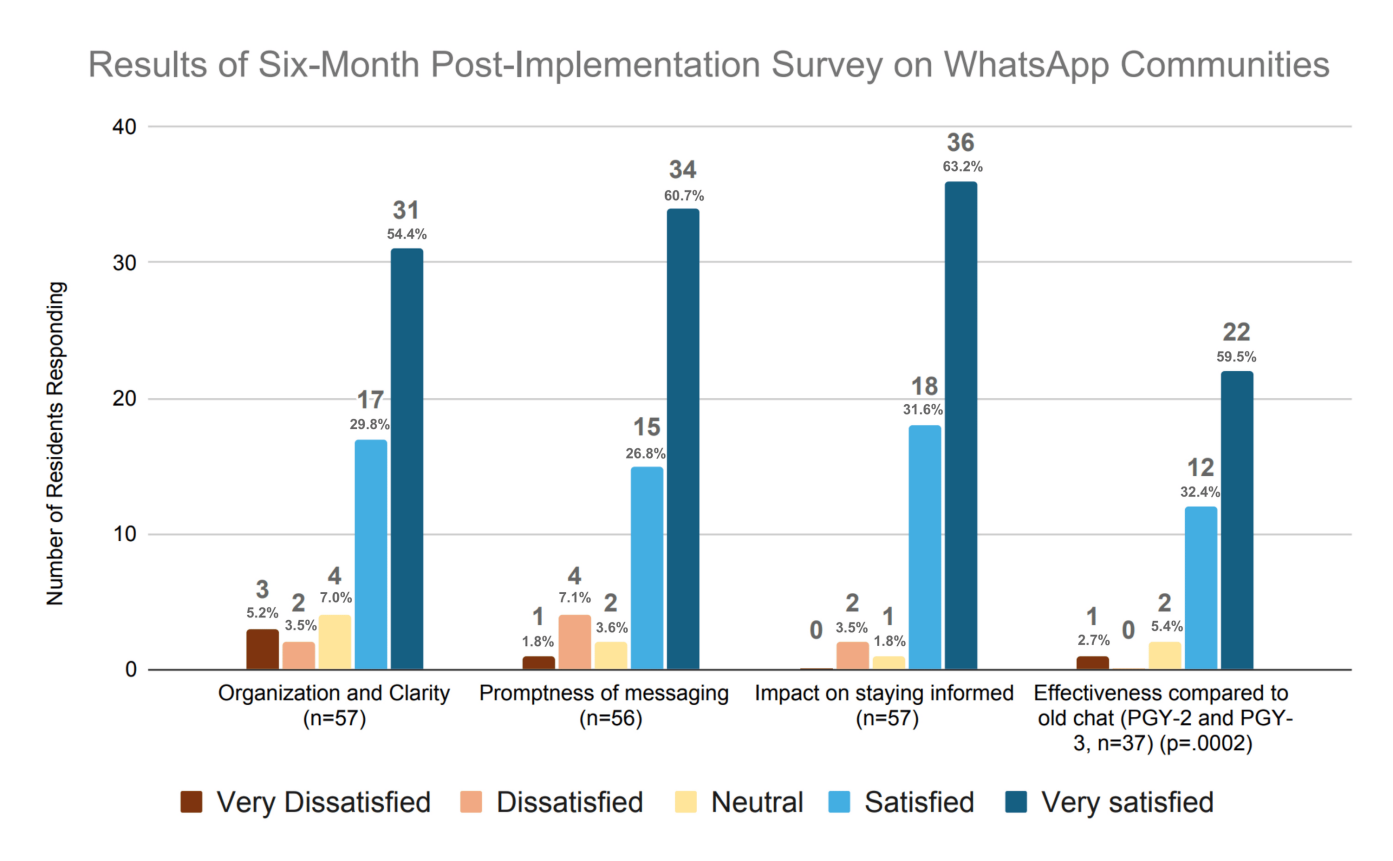
Survey results six months post-WhatsApp Communities implementation.

Of the 120 residents to whom the survey was sent, 57 (47.5%) responded. This included 20 respondents from the PGY-1 class, 21 from PGY-2, and 16 from PGY-3. Results revealed overwhelmingly positive resident sentiment toward WAC as the program’s communication platform: 48 (84%) residents were satisfied with its organization, 49 (87.5%) with the promptness of messaging, and 54 (95%) felt it effectively kept them informed. When asked to compare the overall efficacy of the newly instated WAC to the previous legacy single group chat, 34 (92%) PGY-2 and PGY-3 respondents endorsed satisfaction. The full survey results are depicted below in Figure 3.

In the free-text question where residents were invited to share feedback, responses were again mostly positive. One resident wrote that “[I] was hesitant to use the new chat community, but it has improved communication especially on the floors and for general messages and alerts to communicate with everyone in the program. Very helpful!” Another responded that the WAC was “[a] great method for communication! Made our work life easier! Thank you!”

When comparing objective "undifferentiated patient-care notification" counts over equivalent 90-day periods between the legacy single group chat and the WAC system, the results again demonstrated that the new system was very effective in reducing these burdensome messages. In the WAC system, there were only six instances where a message meant for a specific resident or cohort was errantly sent to all residents. In contrast, during the same time period one year earlier, when the legacy chat was in use, there were 204 such occurrences. This represents a 97% reduction in "undifferentiated patient-care notifications" with the implementation of the WAC.

**Figure 4:**
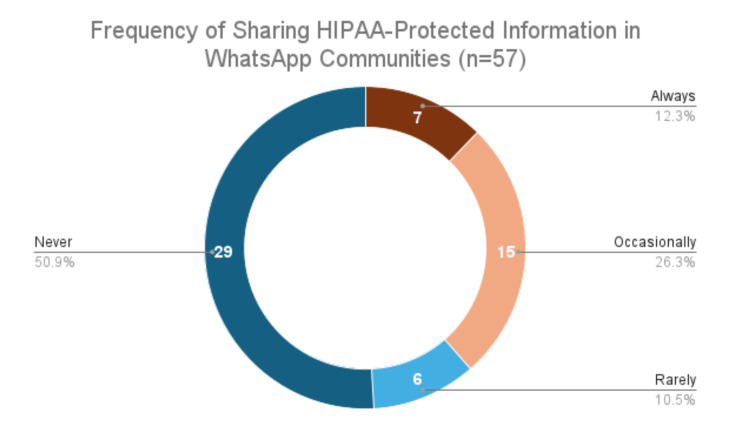
Residents’ self-reported frequency of sharing HIPAA-protected information in WhatsApp Communities. HIPAA, Health Insurance Portability and Accountability Act

The final aspect of our assessment examined how often residents shared PHI on the WAC. When asked to self-report, 29 (51%) stated they never shared PHI, while 28 (49%) admitted to doing so, whether rarely, occasionally, or always (Figure 4).

As alluded to in the Methods section, these results triggered the implementation of an intervention designed to eliminate PHI sharing in the WAC. We measured instances of PHI shared on the WAC during the week prior to the intervention in each of the internal medicine ward group chats for their respective hospital rotation site (Table 1). While only a fraction of the total messages sent were directly related to patient care, an inappropriately high percentage contained PHI (whether the patient’s name or DOB). After the intervention, we noticed a decrease in both the total instances as well as the ratio of patient care messaging including sensitive PHI (Table 2).

**Table 1:** Tally of all messages in the WhatsApp Community during the seven days prior to the intervention aimed at reducing PHI sharing. PHI, protected health information

WhatsApp Communities (7 days pre-intervention)			
	Total # of messages	Patient care messages	Messages containing PHI
Hospital A	54	10	9
Hospital B	77	19	12
Hospital C	17	10	5
Hospital D	8	4	3

**Table 2:** Tally of all messages in the WhatsApp Community during the seven days following the intervention aimed at reducing PHI sharing. PHI, protected health information

WhatsApp Communities (7-day post-intervention)			
	Total # of messages	Patient care messages	Messages containing PHI
Hospital A	63	21	13
Hospital B	44	12	2
Hospital C	33	8	2
Hospital D	20	5	0

We then conducted a pooled analysis including all four group chats with both the two-proportion z-test and Fisher’s exact test (Table 3). This analysis showed that our intervention, consisting of CMRs orienting residents on avoiding PHI sharing and individually contacting those who had shared it, led to a statistically significant reduction in inappropriate PHI sharing on the WAC.

**Table 3:** Statistical significance testing on the intervention results. *Statistically significant at p<0.01. PHI, protected health information

Comparison	Pre-intervention (messages containing PHI/all patient care messages)	Post-intervention (messages containing PHI/all patient care messages)	Two-prop z-test, p-value	95% CI (z-test)	Fisher’s exact test, p-value	95% Cl (Fisher)
Pooled (All Groups)	29/43 (0.67)	17/46 (0.37)	0.0021*	(0.129, 0.570)	0.0017*	(1.65, 17.89)

## Discussion

The implementation of WAC as the communication platform for our internal medicine residency program has been a clear success, with an overwhelming majority of residents reporting satisfaction with the new system. A potential limitation of the study was in the total number of respondents; however, it still constituted a representative sample given adequate representation of each class (PGY-1, PGY-2, and PGY-3).

WAC’s organized, compartmentalized structure, coupled with the ability to selectively enable notifications for active rotations, has significantly reduced distractions. The platform has almost entirely eliminated "undifferentiated patient-care notifications," which previously interrupted residents during clinical duties and often led them to mute all notifications, causing them to miss any subsequent critical updates.

Allowing residents to mute non-essential notifications while continuing to be notified about messages relevant to their current rotation ensures that communication is channeled to intended recipients without distracting or overwhelming others. Over the course of training, even the few seconds spent to view and dismiss irrelevant messages can accumulate significantly. WAC’s ability to reduce these distractions is expected to save residents meaningful time over the course of their training as well as to enhance their overall wellness.

The improvements in organization, timely communication, and staying informed in a fast-paced clinical environment all point to enhanced resident efficiency and satisfaction. That alone makes WAC an attractive model for other residency programs. The fact that these benefits are achieved using a free, easily accessible platform makes the impact even more meaningful, offering a low-effort, high-yield approach to improving communication and promoting resident wellness.

One challenge we encountered was that nearly half of the surveyed residents admitted to sharing PHI on the WAC. As referenced in the introduction, this concern has frequently been raised in the literature regarding the use of WhatsApp and other non-HIPAA compliant messaging apps among healthcare professionals [7]. Our secondary intervention involving CMRs orienting residents on inappropriate usage of PHI and reaching out to residents individually significantly reduced the inappropriate sharing of PHI after a single cycle. Additional interventions and/or regular monitoring must be undertaken to ensure HIPAA compliance.

During the course of our WAC implementation, we identified and implemented an additional strategy to protect against some of the consequences of improper PHI sharing. WAC administrators set a function to automatically erase all messages after seven days. Per WhatsApp’s policy, after that interval has passed, all chat data is erased from both users’ phones as well as their central servers [16]. While this feature does not equate to HIPAA compliance, it does reduce the risk of harm when residents fail to appropriately screen their messages for PHI.

Existing literature on the use of WhatsApp in healthcare primarily focuses on its benefits and drawbacks in clinical practice. There is limited research available on the use of WhatsApp in graduate medical education, particularly its use as a platform for collaboration. Our quality improvement project is distinct in explicitly evaluating the use of WAC as a communication platform within a residency program.

## Conclusions

WAC is a recently launched feature within WhatsApp that allows for collaboration among large groups using multiple, structured sub-groups. Its ability to selectively direct communication to specific individuals or cohorts within a larger group makes it an ideal tool for residency programs, where residents often work across multiple clinical sites simultaneously. WAC’s accessibility and zero cost make it an attractive solution for programs seeking to improve communication and collaboration despite limited resources.

One major challenge is the risk of inadvertent sharing of PHI, given that WhatsApp is not HIPAA compliant. Our secondary intervention showed that this risk can be mitigated by regular reminders from CMRs that reorient residents on the appropriate sharing of sensitive information. By doing so, CMRs can help foster a program culture prioritizing PHI protection, allowing residents to fully benefit from WAC’s strengths while minimizing potential harm.
